# Preventive and clinical care provided to adolescents attending public oral health services New South Wales, Australia: a retrospective study

**DOI:** 10.1186/1472-6831-14-142

**Published:** 2014-11-28

**Authors:** Angela V Masoe, Anthony S Blinkhorn, Jane Taylor, Fiona A Blinkhorn

**Affiliations:** Faculty of Health, School of Health Sciences, Oral Health, University of Newcastle, Ourimbah, NSW 2258 Australia; Population Oral Health Unit, Faculty of Dentistry, University of Sydney, 1 Mons Road, Westmead, NSW 2145 Australia

**Keywords:** Adolescents preventive care, Dental therapists, Public oral health service

## Abstract

**Background:**

Dental Therapists and Oral Health Therapists (Therapists) working in the New South Wales (NSW) Public Oral Health Service are charged with providing clinical dental treatment including preventive care for all children under 18 years of age. Adolescents in particular are at risk of dental caries and periodontal disease which may be controlled through health education and clinical preventive interventions. However, there is a dearth of evidence about the type or the proportion of clinical time allocated to preventive care.

The aim of this study is to record the proportion and type of preventive care and clinical treatment activities provided by Therapists to adolescents accessing the NSW Public Oral Health Service.

**Methods:**

Clinical dental activity data for adolescents was obtained from the NSW Health electronic Information System for Oral Health (ISOH) for the year 2011. Clinical activities of Therapists were examined in relation to the provision of different types of preventive care for adolescents by interrogating state-wide public oral health data stored on ISOH.

**Results:**

Therapists were responsible for 79.7 percent of the preventive care and 83.0 percent of the restorative treatment offered to adolescents accessing Public Oral Health Services over the one year period. Preventive care provided by Therapists for adolescents varied across Local Health Districts ranging from 32.0 percent to 55.8 percent of their clinical activity.

**Conclusions:**

Therapists provided the majority of clinical care to adolescents accessing NSW Public Oral Health Services. The proportion of time spent undertaking prevention varied widely between Local Health Districts. The reasons for this variation require further investigation.

## Background

The Public Oral Health Service in NSW is charged with providing both clinical dental treatment and preventive care [1]. Oral health care for children under 18 years of age in the NSW Public Oral Health Service is mainly provided by Dental Therapists and Oral Health Therapists (Therapists) and has prevention as one of their key performance indicators [2, 3]. The term Therapists will be used for both groups hereafter. There is little information on how Therapists allocate their clinic time when providing dental and preventive care to adolescents.

Adolescents (12 to 18 years of age) are a well-defined population group for whom dental ill health can be a problem [4, 5]. These patients are identified as having distinctive needs due to their tendency for inappropriate dietary habits; likelihood of a high caries rate; use of tobacco, alcohol and other drugs, eating disorders, potential increase of gingivitis leading to periodontal disease and unique social and psychological needs [6, 7]. Dental caries is the most common health problem for adolescents [5, 6, 8]. Researchers report that children with caries are more likely to experience dental caries as adults, with patterns of dental caries changing from a swift developing problem of childhood to a gradual progressive disease of adulthood [9], hence, it is pivotal for Therapists to seize opportunities to offer preventive oral health care and individual support towards self-efficacy in the clinical setting during the adolescence years; this may yield better oral health outcomes for these patients [10–12].

The oral health of the Australian population has improved over the last decades but not uniformly [5, 13, 14]. The NSW Teen Dental Survey 2010 reported a mean DMFT of 1.2 for 14 and 15 year olds in NSW, with the Mid North Coast region having the highest mean DMFT of 3.0 and Hunter New England having the lowest mean DMFT of 0.5 [14]. Although the Teen Survey authors reported limitations in gaining a true representative sample across NSW, the mean DMFT scores for 14 and15 year olds over the last decade have remained fairly stable [14]. Nevertheless, several groups within the population sampled experienced higher burdens of disease; those in rural and remote geographical locations in NSW, limited access to fluoridated water supplies, low socio-economic status and low household income [4, 5, 14, 15]. For example the reported mean DMFT for NSW adolescents from rural and remote regions was 2.4 compared to that in the major cities of 1.2 [14]. Those individuals that usually present to the Public Oral Health Service for dental treatment would benefit greatly from preventive oral health care and advice (9).

Therapists in Public Oral Health settings are well placed to engage and support adolescent’s self-efficacy towards sound oral health, underpinned by the Common Risk Factor Approach principles [16, 17] and aligned with the NSW Healthy Kids Initiatives and Oral Health 2020 [1, 18]. Therapists have the opportunity to offer effective levels of evidence based preventive care, such as topical fluorides and fissure sealants [1, 9] to prevent and control dental caries and provide oral hygiene instruction to improve gingival health and promote use of fluoride toothpaste twice a day [9, 19] to combat dental disease for patients regularly attending public dental clinics [20]. Additionally, with parental influence waning, Therapists as primary health providers may use Motivational Interviewing techniques to guide adolescents towards improving their individual oral health care habits [21, 22]. This is in line with the preventive philosophy of the Australian Commonwealth Government Medicare Teen Dental Program for eligible adolescents introduced in 2008 [23].

All adolescents in NSW are eligible for free oral health care until their eighteenth birthday, however priority is given to those reporting highest dental need (pain) during telephone triage [24]. The NSW Ministry of Health has overarching key governing policies for emergency (pain relief); restorative treatment [24] and three specific preventive care policies for children under 18 years of age for clinical staff working in the Public Oral Health Service which are:

(i) To provide fluoride treatments and fluoride toothpaste advice [25].
(ii) To place pit and fissure sealants [26].
(iii) To offer Brief Intervention Smoking Cessation at the Chairside [27].

There is little information about how effective the Policy Directives are in persuading Therapists to embed these items of preventive care for adolescents into their clinical practice. It is important to monitor the Policies, as if implemented they could greatly improve the oral health of their adolescent patients. However, if preventive care is not being offered it is important to determine the reasons why the Policy is not being implemented.

This retrospective study was undertaken to examine the clinical activities of Therapists in relation to the provision of preventive care for adolescents by interrogating state-wide Public Oral Health Service Electronic Health Record (EHR) data stored on the Information System for Oral Health (ISOH).

## Methods

Items of clinical treatment and preventive care provided by Therapists to adolescents (12 to 18 years of age) in NSW for the financial year 2010/11 were collected from the Information System for Oral Health (ISOH) managed by the Centre for Oral Health Strategy, NSW Health. ISOH is the main repository that stores all clinical patient data for Therapists employed by NSW Public Oral Health Service. Clinical activity was identified by dental treatment item numbers based on The Australian National Dental Schedule System [28]. The data were provided for each Local Health District by activity type and age group. The items were further grouped according to item description (e.g. examinations, restorative and topical fluoride item numbers) and classified into two categories:

(i) Diagnostics and Clinical Treatment (oral examinations, diagnostic tests, radiographs, restorations and extractions).
(ii) Preventive Care (dietary advice, oral hygiene instruction, professional cleaning (i.e. plaque and calculus removal), topical fluoride applications, fissure sealants and smoking cessation).

Radiographs are incorporated in the age preventive category results to illustrate its necessity as a component of the preventive clinical activity for oral disease management processes.

The data were analysed using the Statistical Product and Service Solution V21 (SPSS) [29]. Percentages were used to describe key findings.

Ethical approval for the study was obtained from the Hunter New England Lead Health and Research Ethics Committee (HREC) Reference No. 12/02/15/5.04 and each of the fifteen Local Health Districts (LHDs). The Chief Medical Officer, NSW Ministry of Health approved the use of data from ISOH for this research.

## Results

The ISOH data showed that Therapists provided 79.7 percent of the clinical preventive activities and the majority of restorative treatment (83.0%) for adolescents who attended Public Oral Health Service clinics during 2010/11 with the remaining care undertaken by dentists, specialists and students (Table 1). In that year, Therapists provided dental care for 29, 599 adolescents who accessed NSW Public Oral Health Services, approximately 5.5 percent of NSW eligible adolescent population [30].

**Figure 1:**
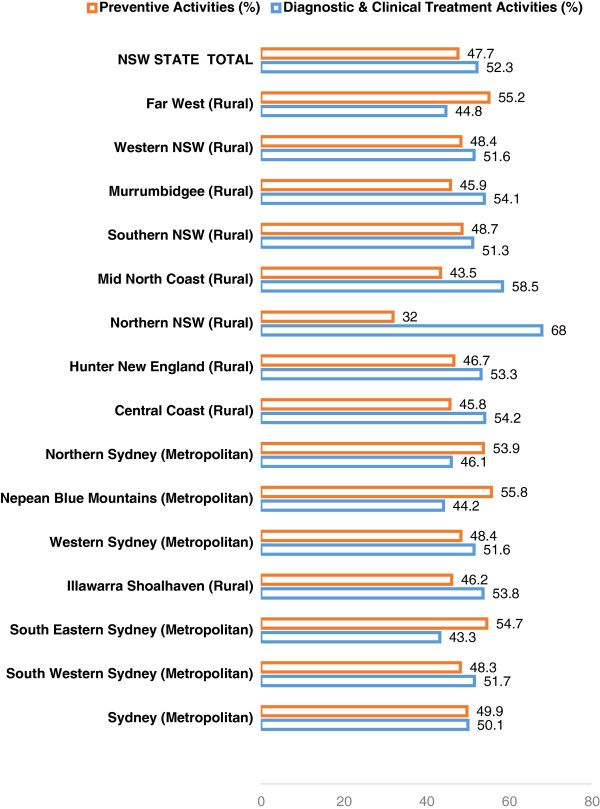
**Local health district’s therapists preventive and clinical activities performed for adolescents, year 2011.**

The proportion of preventive care offered to adolescents varied widely across the State of NSW, from 32.0 percent in Northern NSW LHD to 55.8 percent in Nepean Blue Mountains LHD (Figure 1). Therapists from four LHDs provided preventive care in excess of 50 percent of their clinic time; Nepean Blue Mountains 55.8 percent, Far West 55.2 percent, South Eastern Sydney 54.7 percent and Northern Sydney 53.9 percent. The only LHD which recorded preventive activities below 40 percent was Northern NSW LHD which includes the shires of Ballina and Byron Bay (Figure 1).

**Figure 2:**
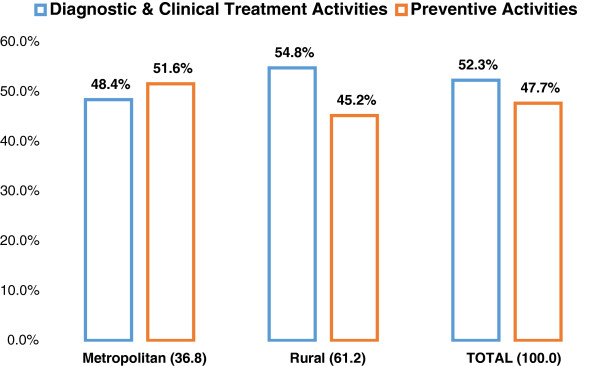
**Percentages of therapists preventive and clinical care activities performed for adolescents in New South Wales metropolitan, rural and remote regions, year 2011.**

Overall, less preventive care (47.7%) was undertaken than diagnostic and clinical treatment (52.3%). Rural and remote LHDs undertook less preventive care (45.2%) compared with metropolitan counter parts (51.6%) (Figure 2).

**Table 1:** **Preventive and restorative weighted occasions of service for adolescents according to class of clinical practitioner, 2010/11**

Practitioner	Preventive		Restorative	
	N	%	N	%
Therapists	32, 292	79.7	18, 620	83.0
Dentists	5, 545	13.7	3, 017	13.5
Specialists & University Students	2, 661	6.6	860	3.5
**TOTAL**	**40, 498**	**100**	**22, 497**	**100**

Time given to dietary advice activities varied across the LHDs from 3.2 percent in Nepean Blue Mountains LHD to 17.8 percent in Western Sydney LHD. Overall, rural areas provided lower levels of dietary advice (below 10%) compared to metropolitan areas (Table 2).

**Table 2:** **Number and percentage (within Local Health Districts) of therapists preventive and clinical activities performed for adolescents, year 2011**

Local Health District	Radiographs	Dietary advice	Oral hygiene instruction	Professional clean	Topical fluoride	Fissure sealant	Smoking cessation	Restorative	Extraction	TOTAL
	N (%)	N (%)	N (%)	N (%)	N (%)	N (%)	N (%)	N (%)	N (%)	N (%)
**Sydney (Metropolitan)**	1888 (23.0)	785 (9.6)	1105 (13.5)	985 (12.0)	668 (8.1)	1373 (16.7)	10 (0.10)	1263 (15.4)	137 (1.7)	**8214** (100.0)
**South Western Sydney (Metropolitan)**	3936 (21.9)	1889 (10.5)	2552 (14.2)	1136 (6.3)	3455 (19.2)	2015 (11.2)	9 (0.10)	2459 (13.7)	518 (2.9)	**17969** (100.0)
**South Eastern Sydney (Metropolitan)**	1626 (12.0)	1454 (13.0)	1477 (13.2)	1582 (14.2)	2137 (19.2)	782 (7.0)	11 (0.1)	1869 (16.7)	222 (2.0)	**11160** (100.0)
**Illawarra Shoalhaven (Metropolitan)**	2506 (18.0)	1674 (12.0)	2213 (15.9)	1740 (12.5)	1309 (10.0)	1116 (8.0)	10 (0.1)	2820 (20.3)	453 (3.3)	**13922** (100.0)
**Western Sydney (Metropolitan)**	2124 (23.4)	1622 (17.8)	1945 (21.4)	759 (8.4)	571 (6.2)	750 (8.3)	21 (0.2)	1005 (11.1)	290 (3.2)	**9087** (100.0)
**Nepean Blue Mountains (Metropolitan)**	2126 (13.2)	434 (3.2)	1294 (9.6)	1315 (9.8)	4340 (32.3)	1585 (11.8)	9 (0.1)	1916 (14.2)	452 (3.4)	**13471** (100.0)
**Northern Sydney (Metropolitan)**	1058 (18.0)	592 (10.0)	1050 (17.9)	743 (12.7)	772 (13.2)	845 (14.4)	2 (0.0)	698 (11.9)	105 (1.8)	**5865** (100.0)
**Central Coast (Rural)**	3426 (25.5)	1619 (12.0)	1799 (13.4)	504 (3.7)	2672 (19.9)	1084 (8.1)	25 (0.2)	2034 (15.1)	281 (2.1)	**13444** (100.0)
**Hunter New England (Rural)**	7170 (24.4)	1086 (3.7)	4448 (15.1)	2354 (8.0)	4161 (14.1)	5507 (18.7)	51 (0.2)	3847 (13.1)	784 (2.7)	**29408** (100.0)
**Northern NSW (Rural)**	1596 (21.9)	452 (6.2)	1251 (17.2)	399 (5.5)	393 (5.4)	685 (9.4)	12 (0.2)	2246 (30.9)	240 (3.3)	**7274** (100.0)
**Mid North Coast (Rural)**	1885 (21.2)	431 (4.8)	1257 (14.1)	781 (8.8)	1345 (15.1)	1097 (12.3)	3 (0.0)	1853 (20.8)	259 (2.9)	**8911** (100.0)
**Southern NSW (Rural)**	1113 (16.7)	309 (4.6)	669 (10.1)	526 (7.9)	904 (13.6)	1733 (26.1)	35 (0.5)	1174 (17.7)	188 (2.8)	**6651** (100.0)
**Murrumbidgee (Rural)**	1720 (14.8)	604 (5.2)	1724 (14.8)	1224 (10.5)	1385 (11.9)	1889 (16.2)	38 (0.3)	2676 (23.0)	391 (3.4)	**11651** (100.0)
**Western NSW (Rural)**	1547 (20.0)	386 (5.0)	1334 (17.2)	396 (5.1)	619 (8.0)	1801 (23.3)	9 (0.1)	1416 (18.3)	236 (3.0)	**7744** (100.0)
**Far West (Rural)**	214 (14.0)	161 (10.5)	269 (17.6)	182 (11.9)	358 (23.4)	97 (6.4)	1 (0.1)	212 (13.9)	33 (2.2)	**1527** (100.0)
**TOTAL NSW**	**33935 (20.4)**	**13498 (8.1)**	**24387 (14.7)**	**14626 (8.8)**	**25170 (15.2)**	**22359 (13.4)**	**246 (0.1)**	**27488 (16.5)**	**4589 (2.8)**	**166298 (100.0)**

There were differences between LHDs in the proportion of time spent offering oral hygiene instruction. This ranged between 10.1 percent (Southern NSW) to 21.4 percent (Western Sydney) (Table 2). Professional cleaning for adolescents also differed between LHDs ranging from 5.1 percent in Western NSW to 14.2 percent in South Eastern Sydney. There was a wide variation in the application of topical fluoride from 5.4 percent in Northern NSW LHD to 23.4 percent in the Far West (Table 2).

Table 2 also shows a wide variation in the use of fissure sealants in rural Local Health Districts, ranging from 12.3 percent for Mid North Coast to 26.1 percent for Southern NSW. Conversely, in metropolitan LHDs the proportion of fissure sealants placed ranged from 7 percent in South Eastern Sydney to 16.7 percent for Sydney LHD.

Although brief smoking cessation advice is a NSW Health Policy, Table 2 shows that it was rarely offered, but did increase with the age of the patient being treated (Table 3).

Clinical restorative activities ranged from 11.1percent for adolescents in Western Sydney to a high of 30.9 percent provided for patients in Northern NSW LHD. There was less variation with the proportions of dental extractions, ranging from a high of 3.4 percent for both Murrumbidgee and Nepean Blue Mountains to a low of 1.7 percent in Sydney LHD. There was also a notable variation in the number of radiographs taken ranging from 14.0 percent in Far West to 25.5 percent in the Central Coast (Table 2).

There was little difference in the percentage of preventive clinical activities undertaken within age groups, the exceptions being smoking cessation advice which increased with patients’ age and the placement of fissure sealants which peaked at 13–14 years (15.2%). Radiographs as a baseline diagnostic and caries management tool increased steadily with age advancement, with a slight decrease at age 17 (18.8% to 21.8) (Table 3).

**Table 3:** **Number and percentages (within age) of therapists preventive clinical activities performed for adolescents, year 2011**

Preventive activity	Age						Total
	12	13	14	15	16	17	
	N (%)	N (%)	N (%)	N (%)	N (%)	N (%)	N (%)
Dietary Advice	3032 (9.2)	2616 (8.2)	2287 (7.8)	2064 (7.9)	1784 (7.7)	1724 (7.5)	13498 (8.1)
Oral Hygiene Instruction	5381 (16.4)	4801 (15.1)	4217 (14.3)	3751 (14.3)	3204 (13.8)	3033 (13.3)	24387 (14.7)
Professional Clean (plaque and calculus removal)	2884 (8.8)	268 (8.5)	2540 (8.6)	2297 (8.8)	2041 (8.8)	2177 (9.5)	14626 (8.8)
Topical Fluoride Application	4940 (15.0)	4878 (15.4)	4378 (14.9)	3941 (15.0)	3571 (15.4)	3462 (15.2)	25170 (15.2)
Fissure Sealant	4578 (13.9)	4745 (15.0)	4470 (15.2)	3502 (13.4)	2590 (11.2)	2474 (10.8)	22359 (13.4)
Radiographs	6191 (18.8)	6238 (19.7)	6059 (20.5)	5489 (20.9)	5038 (21.8)	4920 (21.5)	33935 (20.4)
Smoking Cessation	0 (0.0)	6 (0.0)	26 (0.1)	43 (0.2)	69 (0.3)	102 (0.4)	246 (0.1)
**NSW STATE TOTAL**	**32856 (100.0)**	**31739 (100.0)**	**29502 (100.0)**	**26205 (100.0)**	**23161 (100.0)**	**22835 (100.0)**	**166298 (100.0)**

The offer of dietary advice across the age groups was consistently below 10.0 percent, declining with age of the patient to 7.5 percent for 17 year olds. Oral hygiene instruction followed the same pattern with 16.4 percent provided for 12 year olds, dropping to 13.3 percent for 17 year olds (Table 3). Professional cleaning (removal of plaque and calculus) activities to support and maintain healthy gingivae for adolescents were less than 10 percent across all age groups (Table 3).

Topical fluoride was rarely offered with percentages fluctuating around 15 percent across all age groups (Table 3).

## Discussion

All adolescents in NSW are eligible for free public oral health care provided through primary community health, hospital and school settings by Therapists [24]. This care is enhanced by an established consultative and collaborative working relationship with dentists and Paediatric Dental Specialists. The Australian Commonwealth Government Medicare Teen Dental Program in response to concerns raised regarding adolescents being at risk of dental disease, offered adolescents whose families are eligible for Family Tax A, a preventive voucher that could be used in private and public dental services [23]. However, there were problems with this preventive scheme, as some eligible adolescents seeking private care were referred back to the public system when their parents were unable to meet the on-going costs of treatment outside the parameters of the voucher [14]. Most adolescents accessing NSW Public Oral Health Services are from disadvantaged groups including the working poor. These individuals would benefit greatly from preventive care and advice [10, 12, 21, 31, 32] from Therapists.

### Fluoride policy

The NSW fluoride policy pertaining to professionally applied fluoride products for individuals above 10 years of age recommends that fluoride varnish and/or high concentrated fluoride gels should be used for patients who have an elevated risk of developing caries [25]. Topical fluoride use has been scientifically proven to be effective in the prevention and control of dental caries and their use for caries stabilisation [9, 33, 34].

The majority of adolescents being treated came from deprived (low socio-economic) and rural and remote areas where caries rates are high [14]. For example the high levels of restorative and extraction activities recorded for Northern NSW (Table 2) which is an unfluoridated area should signpost the importance of providing fluoride treatments for these at risk patients. However, the Therapists recorded an actual level of only 5.4 percent topical fluoride treatments for these patients. Adolescents living in Northern NSW have high levels of dental caries and Therapists from this area did spend time on oral hygiene instruction (17.2%) and the use of fluoride tooth paste, which aligns with the fluoride policy [25]. Therapists in two other LHDs spent similar proportion of time on oral hygiene instruction, one metropolitan and one rural. The reasons other LHDs spent less time is not clear, and further research using qualitative approaches is warranted.

Skinner et al. [14] reported a mean DMFT of 2.4 for NSW rural and remote regions, with a mean DMFT for Mid North Coast of 3.0 therefore provision of topical fluoride treatments for adolescents residing in these areas is especially important in conjunction with oral hygiene education and promotion of tooth brushing with fluoride toothpaste, according to government policy [14, 25]. This study illustrated fluoride applications for rural and remote areas varied between 5.4 percent (Northern NSW) to 23.4 percent for Far West LHD, compared to metropolitan LHDs ranging from 8.1 percent (Sydney) to 32.3 percent in Nepean Blue Mountains.

Considering the Australian Government Teen Dental Program aimed at low socio-economic families had an emphasis on preventive care [23], it is disappointing to note the low levels of topical fluoride use and oral hygiene instruction across LHDs, as these are relatively simple and quick procedures that assist in dental caries prevention [9]. Previous studies have identified factors influencing clinician’s adherence to preventive guidelines for example lack of time, variances in practitioners awareness of protocols, guidelines and individual habitual clinical behaviours [35–37].

### Pit and fissure sealant: use of in oral health services, NSW policy

Placement of fissure sealants in the occlusal surfaces of permanent molars, the sites most susceptible to dental caries is a proven clinical preventive intervention, especially for those individuals classed as being at high risk of developing dental caries [26, 38].

This study recorded the percentages of fissure sealants provided by Therapists for adolescents accessing the NSW Public Oral Health system; although levels of fissure sealants across LHDs were fairly low, the findings illustrated that Therapists placed fissure sealants aligned with permanent tooth eruption age timeframes. According to Skinner et al. [14] in their State-wide dental survey, South Eastern Sydney LHD 14–15 year olds had the most fissure sealants in their permanent teeth across NSW. However, in our study fissure sealant percentages provided to adolescents by Therapists in this LHD were particularly low. This suggests the focus on fissure sealants as a preventive modality for 14–15 year olds may well have occurred in the private sector. A study undertaken by Clarkson et al. [39] in Scotland offered incentives of remuneration and training for the underutilised practice of placement of fissure sealants by dentists. Following the intervention the authors reported a 9.8% increase in the provision of fissure sealants, with no significant difference noted in the type of education provided [39]. Little is known of the impact the Australian Government Teen Dental Program vouchers had on Therapists fissure sealant preventive practice as separate treatment items were not reported as there was a flat fee per voucher.

The use of bitewing radiographs by Therapists as a diagnostic and caries management tool was clearly not a standard procedure. This is clearly insufficient as they provide relevant clinical information prior to the placement of fissure sealants and planning a preventive strategy [26, 32, 40]. Despite the evidence of the value of fissure sealants as a preventive treatment, most NSW LHDs with high restorative and dental extraction activities reported low fissure sealant placement activity.

Bonetti et al’s [35] study used psychological models to understand and predict general dental practitioner’s clinical behaviour to placement of fissure sealants. The authors suggested that evidence-based behaviour of clinicians can be enhanced by influencing beliefs of the positive outcomes of fissure sealant placements and creating a clinical habit of performing them as an integral part of patient management.

Overall, the provision of fissure sealants as a preventive modality was inadequate in comparison with the time devoted to restorative care across the State for adolescents. Satur et al’s [41] study reported that due to greater demand in rural areas for urgent treatment including emergencies, less preventive care was being offered to patients, and this may explain why some Therapists were not placing fissure sealants.

### Smoking cessation brief intervention at the chairside: role of public oral health/dental services policy

According to the Cancer Council Australia, 80% of adults become addicted to smoking during their adolescent years [42]. Researchers recommend provision of smoking cessation in the dental setting for adolescents as an early intervention strategy [43]. Self-reporting by patients completing their medical history should be used as a trigger by all clinicians in NSW Public Oral Health Services to provide smoking cessation advice [27]. Therapists identified adolescents for smoking cessation advice commencing at age 13 and the numbers given advice slowly increased with patient age. Nonetheless, despite this policy smoking cessation advice was rarely offered. Trotter and Worcester’s [44] study reported lack of resources and patient materials, clinician doubts regarding being effective, lack of confidence to tackle the issues and support patients to quit smoking and insufficient time as barriers to perform preventive activities. Our study suggests that further review and on-going training support for Therapists is required if smoking cessation is going to be offered routinely in line with the NSW Policy.

### Oral hygiene instruction

Adolescence is a critical developmental life stage whereby clinicians may engage with patients to promote self-efficacy towards improved oral health practices for long term health outcomes [21, 31]. In this study the offer of oral hygiene education to adolescents to promote brushing with fluoride toothpaste was low across all LHDs.

Oral hygiene is an individual’s personal maintenance plan to disrupt the plaque biofilm to prevent its accumulation on teeth and gingiva [45]. The promotion of brushing twice a day with a fluoride tooth paste to prevent dental caries is an important part of this activity. Oral hygiene education guidelines for adolescents are lacking, but, toothbrushing with a fluoride toothpaste are part of the NSW Health Fluoride Use Policy [25]. Therefore, Therapists should be supported in the clinical setting with the provision of oral health products (fluoride toothpaste and tooth brush) to issue to patients for home-care use in line with the Ottawa Charter ‘supportive environment’ principle [46]. There was a decline of oral hygiene education for older adolescents which is a concern considering evidence of adolescents’ levels of caries experience [5, 14] and their prospective as young parents.

Studies linking periodontal disease with systemic disease suggest that Therapists should offer professional prophylaxis in association with oral hygiene instruction [47, 48]. This study illustrated that professional cleaning (plaque and calculus removal) clinical activities provided for adolescents could be improved, and this may happen as the proportion of dually qualified Therapists and Hygienists increases.

### Dietary advice

There is overwhelming evidence regarding the role of sugar and its frequent consumption in the aetiology of dental caries [49, 50], however dietary advice was given little time by Therapists in NSW. Somewhat surprisingly, given the higher levels of dental caries this study found that in six rural LHDs, Therapists provided lower levels of dietary advice to adolescents when compared with Therapists in metropolitan LHDs. There is certainly scope for further research to investigate why Therapists offering dietary advice is so variable across LHDs. Therapists in public health settings have opportunities to provide dietary advice for adolescents utilising different strategies such as adopting Motivational Interviewing techniques and utilising easy to translate diet tip sheets [21, 31].

There is a plethora of evidence regarding adolescent’s dietary habits. Parents and health practitioners face fierce media advertising of carbonated beverages, sport drinks and sugary snacks [51–53]. Nevertheless, it is important that Therapists work in collaboration with allied health professionals for example dieticians, diabetic educators, health promotion professionals and local community agencies to offer advice to young people so they have the knowledge to change their behaviour. This aligns with the Common Risk Factor Approach principles [16, 17] so that advice on oral health fits in with the general health concerns of trying to reduce obesity and early onset of diabetes [1, 54] and systemic diseases [47].

This is the first study undertaken in NSW to record the provision of preventive care provided by Therapists to adolescents accessing the Public Oral Health Service. The re-use of the ISOH data for research purposes requires more investigation. A useful first step would be to assess the reliability of the data in more detail. In addition, the way preventive items are coded should be the subject of a review as currently preventive activities are not captured in any great depth and useful data may be missed.

## Conclusions

The retrospective study into the provision of preventive and clinical treatment by Therapists to adolescents accessing the NSW Public Oral Health Service has demonstrated that adolescents were offered preventive care but there was considerable variation between Local Health Districts.

A review of the way NSW Health Policy Directives are implemented and the reasons for non-compliance should be undertaken at a LHD level. Of particular concern is the need to enhance the use of topical fluorides, placement of fissure sealants, and the provision of dietary and smoking cessation advice.
